# Palliative care consultation services in hospitals in the Netherlands: the design of the COMPASS study

**DOI:** 10.1186/s12904-015-0069-0

**Published:** 2015-12-01

**Authors:** Arianne Brinkman-Stoppelenburg, Suzanne Polinder, Yvonne Vergouwe, Agnes van der Heide

**Affiliations:** Department of Public Health, Erasmus Medical Center, University Medical Center Rotterdam, P.O. Box 2040, 3000 CA Rotterdam, The Netherlands

**Keywords:** Palliative care, Palliative medicine, Referral and consultation, Quality of life, Cancer, Costs and costs analysis, Patient satisfaction, Hospitals, Observational study, Longitudinal study

## Abstract

**Background:**

Patients with an advanced incurable disease are often hospitalised for some time during the last phase of life. Care in hospitals is generally focussed at curing disease and prolonging life and may therefore not in all cases adequately address the needs of such patients. We present the COMPASS study, a study on the effe***c***ts and c***o***sts of consultation tea***m***s for ***pa***lliative care in ho***s***pital***s***.

This observational study aims to investigate the use, effects and costs of PCT consultation services for hospitalized patients with incurable cancer in the Netherlands.

**Methods/design:**

The study consists of 3 parts:

1. A questionnaire, interviews and a focus group discussion to investigate the characteristics of PCT consultation in 12 hospitals. PCTs will register their activities to calculate the costs of PCT consultation.

2. Cancer patients for whom the attending physician would not be surprised that they would die within 12 month will be included in a medical file search in three hospitals. Medical records will be investigated to compare care, treatment and hospital costs between patients with and patients without PCT consultation.

3. In the other nine hospitals, we will perform a longitudinal study, and compare quality of life between 100 patients for whom a PCT was consulted with 200 patients without PCT consultation. Propensity score matching will be used to adjust for differences between both patient groups. Patients will be followed for three months after inclusion. Quality of life will be assessed with the Palliative Outcome Scale, the EuroQol-5d and the EORTC-QLQ-C15 PAL. Satisfaction with care in the hospital is measured with the IN-PATSAT32. The cost impact of PCT consultation will also be explored.

**Discussion:**

This is the first multicenter study on PCT consultation in the Netherlands. The study will give valuable insight in the process, effects and costs of PCT consultation in hospitals. It is anticipated that PCT consultation has a positive effect on patients’ quality of life and satisfaction with care and will lead to less hospital care costs.

## Background

Patients with an advanced incurable disease often spend some time in hospital during the last months of life [1]. When death is approaching for such patients, the goals of care need to be realigned. However, burdensome medical interventions are often prolonged until the end of life, without any beneficial effect [2–5]. As a result, end-of-life care in hospitals tends to involve high costs while failing to address patients’ needs and to provide them with a dignified death.

Palliative care is aimed at acknowledging patients’ impending death and at reconsidering goals of care [6]. It is an approach that improves the quality of life and their families facing the problems associated with life-threatening illness, through the prevention and relief of suffering by means of early identification and impeccable assessment and treatment of pain and other problems, physical, psychosocial and spiritual [7]. Palliative care is not only provided at the end of life, but also in earlier stages, sometimes alongside disease-directed interventions that are aimed at curing disease or prolonging life [8]. Basic palliative care, such as basic symptom management and supporting patients to align their treatment choices with their values and goals, should be delivered by any health professional attending patients in the last phase of life. Other, more complex forms of care, such as managing refractory symptoms or negotiating a difficult family meeting, may require involvement of specialized palliative care professionals [8].

### The effects of palliative care team consultation

Several studies have shown that the deployment of specialized palliative care teams (PCTs) in hospitals is associated with better outcomes for patients with advanced disease [9]. Their involvement was found to improve patients’ quality of life [10–12], their satisfaction with care [12, 13] and communication about goals of care, resulting in less diagnostic testing and less use of inappropriate technology and intensive care [14]. In a recent study Temel et al. found that patients with lung cancer receiving early palliative care had a better quality of life, received less aggressive treatment and had a longer survival compared to patients receiving usual care without palliative care involvement [15]. Consultation of palliative care services for patients with advanced incurable disease may also influence health care costs, through its focus on assessing patients’ goals of care and providing treatments that are concordant with these goals. Several studies have demonstrated significant cost savings as a result of palliative care involvement [13, 14, 16–18]. The largest group of patients who are referred to specialized palliative or end-of-life care services consists of patients with incurable cancer, in the Netherlands as well as in other countries [19, 20]. However, the provision of palliative care to patients with advanced cancer often remains suboptimal. Informational, emotional and physical needs are frequently unmet among patients with incurable disease [21–24]. Studies show that PCT consultation is only used for a minority of all patients with advanced disease, which suggests that this service is used sub-optimally [25]. This might be due to a lack of awareness of the availability and potential contribution of this service among regular health care professionals. Furthermore, it is known that PCTs are often consulted late in the disease trajectory [26]. There may also be barriers to consultation, such as the lack of referral criteria, and the view that the involvement of palliative care is a signal that the primary health care professionals have given up all hope for a patient [27–29].

### Palliative care team consultation in hospitals in the Netherlands

The Netherlands has a nationwide system of palliative care consultation services, that is predominantly used by general practitioners for patients staying at home [30–32]. More recently, hospitals have started establishing PCT consultation services. An inventory of the Comprehensive Cancer Centre Netherlands in 2013 showed that at least 45 hospitals in the Netherlands, out of a total of 92, were in different stages of implementing such consultation services [33]. Most hospital-based specialist PCTs can be consulted by physicians or nurses working in the hospital. Upon their involvement, PCTs typically provide a detailed holistic assessment of the patients’ and family´s situation. They assess patients’ symptoms and their physical, emotional, social and spiritual problems, prioritize these and propose a care plan to address them. The extent to which PCTs provide care themselves or only advise the primary caregivers varies. Most PCTs consist of professionals from different specialties, such as oncologists, neurologists, anaesthesiologists, nurses, and psychosocial and spiritual caregivers. The structure and activities of PCTs in hospitals vary. Some PCTs are strongly embedded in the hospital and supported by their hospital boards, whereas others are not. Some teams are involved in education within or outside the hospital.

### Objectives

Evidence on the effects of PCT consultation in hospitals on patient outcomes and health care costs mainly comes from studies from the United States. It is unclear to what extent these results can be generalized to other countries with different health care systems and cultures, such as the Netherlands. We will therefore perform an observational study to investigate the use, effects and costs of PCT consultation services for hospitalized patients with incurable cancer in the Netherlands. We will

1. assess the characteristics of PCT consultation services in different hospitals,
2. study whether PCT consultation for patients with incurable cancer has an effect on in-hospital medical care,
3. explore whether PCT consultation for patients with incurable cancer has beneficial effects on patients’ quality of life and satisfaction with care and
4. explore whether PCT consultation reduces costs of care and might be a cost-effective intervention.

## Methods

We will collect data in three substudies.

### Characteristics of PCT consultation in Dutch hospitals

We will use a structured questionnaire to study structure and process characteristics of PCT consultation services in 12 hospitals in the Netherlands. All teams will be asked to fill out this questionnaire, which contains multiple choice and open questions regarding the disciplines that are represented in the PCT, the process of consultation and the way the teams monitor and improve the quality of their consultation. Further, all teams will be invited to participate in group discussions or individual interviews to explore differences between teams and PCT members’ experiences of barriers and facilitators of a successful consultation service.

### The effect of PCT consultation for patients with incurable cancer on medical care

In three hospitals, care and treatment as provided to patients with advanced incurable cancer for whom a PCT is consulted during their stay in the hospital will be compared with care and treatment as provided to comparable patients without PCT consultation. Inclusion criteria are that patients are 18 years or over and that the attending physician answers “no” to the question “Would you be surprised if this patient would die in the next year?”. We will check the medical files of all eligible patients to assess the use of diagnostic procedures, medication, chemotherapy and other medical interventions, and days spent in hospital, during 3 months after inclusion. The attending physician will be asked to provide information on the patient’s diagnosis, performance status, co morbidity and life expectancy, by filling out a short questionnaire. For those patients for whom a PCT is consulted, PCTs will be asked to fill out a questionnaire about the content of the consultation.

The use of medical care and the number of days spent in hospital will be compared between patients with PCT consultation and patients without PCT consultation, while taking into account differences in patients’ medical characteristics.

### The effect of PCT consultation for patients with incurable cancer on quality of life and the cost effectiveness of PCT consultation

In 9 hospitals we will study the effect of PCT consultation for in-hospital patients with incurable cancer on quality of life. In order to get a good overview of different types of hospitals, a university hospital, teaching and general hospitals will be included. In order to include sufficient comparable patients receiving usual care 3 hospitals without a PCT will be included.

Inclusion criteria are that patients are 18 years or over; that the attending physician answers “no” to the question “Would you be surprised if this patient would die in the next year?”; and that the patient is expected to stay in the hospital for at least 3 days. The latter criterium is added to enable an informed consent procedure. Patients will be included upon arrival in the hospital. Eligible patients will receive an information letter, and will be informed and asked to participate by an attending nurse. Patients who agree to participate will be followed during three months, regardless of where they stay. The attending physician will be asked to fill in a questionnaire to assess medical information such as diagnosis, performance status, co morbidity and life expectancy. Palliative care consultation teams are asked to fill a questionnaire about the content of the consultation. Patients will be asked to fill out a number of subsequent questionnaires. In these questionnaires, quality of life is assessed with the EORTC QLQ-C15 PAL [34]. We will also measure quality of life with the Palliative Outcome Scale [35] and the EuroQol-5D [36] , because of the lack of consensus on the most appropriate quality of life instrument for patients with incurable disease [37]. Secondary measures are symptom scores for pain, dyspnoea and anxiety (EORTC QLQ-C15); patient satisfaction with care, using the INPATSAT 32 questionnaire [38, 39]. Quality of life will be assessed longitudinally at six moments in time: at day 4, 7, 14, 30, 60 and 90 after admission to the hospital; satisfaction with care will be assessed at day 14 after admission.

We aim to include 100 patients for whom a PCT was consulted and compare them with at least 200 patients receiving usual care. With such a sample size we will be able to detect a difference in quality of life as measured by the EORTC QLQ-C15 PAL at day 14 of 0.4 in standard deviation units (Cohen’s D = effect size) with alpha of 0.05 (2-sided) and power of 90 %.

### Data analysis

Due to the observational design of the study it is likely that there is an imbalance of prognostic factors between patients who are and patients who are not receiving PCT consultation. As a consequence the estimated effects of PCT consultation can be biased. We will adjust for imbalance in the statistical analysis by using propensity scores [40–42].

We will develop a propensity score model to assess the probability that a patient would have been offered PCT consultation. Patient characteristics to be included in this model are age, sex, marital status, primary diagnosis, reason for hospitalization (e.g. treatment of complex symptoms, palliative chemotherapy, treatment of complications), life expectancy, functional status, attending physician specialty, comorbidity, and hospital of admission [14, 16]. The logit of the propensity score will be used to match each patient receiving PCT consultation with one or more usual care patients.

Quality of life scores at different time points will be compared between the PCT and care as usual groups with repeated measurement analysis. A model will be fitted that includes matched-pairs, time, treatment group, the baseline score, and interaction between time and treatment group.

### Economic evaluation

The economic evaluation of PCT consultation in hospitals will concern a period of three months after admittance of patients to the hospital.

Data from substudy 2 will be used to perform a cost-minimisation analysis including hospital costs. We will distinguish intramural medical costs (inpatient days, professional health caregivers’ activities, medical procedures). Real medical costs will be calculated by multiplying the volumes of health care use with the corresponding unit prices. Costs for inpatient days in hospitals will be estimated as real, basic costs per day using detailed hospital administrative information. We will make a distinction between the costs in general hospitals and university hospitals. The unit price of the PCTs will be determined with the micro-costing method [43], which is based on a detailed assessment of all resources used.

Data from substudy 3 will be used to perform an economic evaluation from a health care perspective. We will calculate total medical costs per patient, including intramural and extramural medical costs (home care, nursing home days, general practitioner activities). For the calculation of the intramural medical care costs and the unit price of PCTs we use the same methodology as for the cost-minimisation study. For the calculation of extramural medical costs, we will use charges as published in Dutch guidelines as a proxy of real costs [44]. The cost-effectiveness of PCT consultation will be assessed by calculating the incremental cost-effectiveness ratio (ICER), defined as the difference in costs of PCT consultation compared to usual care, divided by the average change in patients’ quality of life . The primary effect measure for the economic evaluation of PCT consultation is quality of life as measured by the EORTC QLQ-C15. Because of the short time horizon, costs and effects will not be discounted.

### Feasibility of recruitment

We expect to be able to include the needed number of patients in a period of 18 months. The participating departments will be asked to assign a nurse who is responsible for checking the eligibility of all admitted patients. This method proved to be successful in a previous study [3]. Patients with incurable cancer have been found to be at least as willing to participate in scientific research as patients in other stages of disease [3, 45]. Whereas our study is an observational study in which patients are only asked to fill out a number of questionnaires, our modest assumption of a 60 % participation rate seems justified.

### Ethical considerations

The research protocol was submitted to the Medical Ethical Research Committee of the Erasmus Medical Center who declared that there were no objections to the performance of this study.

Substudy 1 is not related to patients but to caregivers. Caregivers consented to participate in the group discussions or individual interviews. For substudy 2, according to national regulations, informed consent does not have to be obtained, because data on medical care and decision making will be gathered locally and stored anonymously in a database. For substudy 3, written informed consent will be obtained from all participants.

## Discussion

The objective of the COMPASS study is to assess the process and outcomes of hospital palliative care consultation in the Netherlands. The study has several potential strengths, weaknesses, threats and opportunities that are associated with the design of the study and with the specific population.

### Strengths and weaknesses

To our knowledge this is the first study to assess the effects of hospital palliative care consultation in the Netherlands. The number of participating hospitals [12] and PCTs [9], representing academic, teaching and generals hospitals, is a strength of this study because in this way we gain insight in different settings.Furthermore, we will study PCT consultation in ‘real life’ and not in an experimental setting. However, the observational design of the COMPASS study can also be viewed as a weakness. Randomized studies on the effects of health service changes in palliative and end-of-life care are often not possible, due to ethical and practical constraints. Firstly, specialized palliative care services may not only have an impact on individual patient care, but also on general attitudes of hospital caregivers towards care for patients in the last stage of life, which would contaminate assessments in the control groups. Secondly, several hospitals, especially those that are at the forefront of developing palliative care have already implemented PCT consultation facilities for several years, which makes randomized evaluation of such facilities impossible. When randomized trials are not feasible, as is the case in our study, a well-designed observational study is an appropriate alternative [46]. Some previous studies on the effects and costs of specialized palliative care services were observational and used novel statistical techniques, such as propensity scores, to define comparable patient groups [22].

### Threats and opportunities

The threats to the successful conduct of this study relate mainly to the inclusion of patients. Nurses may be reluctant to ask patients for informed consent as participation in research may impose undue burden on patients and caregivers [47]. However, studies have found that many patients with advanced illnesses actually appreciate to participate in research [47]. Furthermore, it is known that PCTs are often consulted relatively late in the disease trajectory [27, 48], which means patients may not be able to participate by filling in questionnaires. Furthermore, some patients will be lost to follow up, which is inherent to the study population.

### Implications

This study will provide insight in the process and outcomes of PCT consultation in Dutch hospitals. This knowledge is important for existing and new PCTs. The outcomes can be used to optimize hospital-based PCT consultation services for patients with advanced illnesses.

## Abbreviations

PCT: Palliative Care Team

## References

[1] Abarshi E, Echteld M, Van den Block L, Donker G, Deliens L, Onwuteaka-Philipsen B (2010). Transitions between care settings at the end of life in the Netherlands: results from a nationwide study. Palliat Med.

[2] Veerbeek L (2008). Care and quality of life in the dying phase.

[3] Voogt E (2006). Living till the end.

[4] Teno J, Lynn J, Wenger N, Phillips RS, Murphy DP, Connors AF (1997). Advance directives for seriously ill hospitalized patients: effectiveness with the patient self-determination act and the SUPPORT intervention. SUPPORT Investigators. Study to Understand Prognoses and Preferences for Outcomes and Risks of Treatment. J Am Geriatr Soc.

[5] Middlewood S, Gardner G, Gardner A (2001). Dying in hospital: medical failure or natural outcome?. J Pain Symptom Manage.

[6] Sepulveda C, Marlin A, Yoshida T, Ullrich A (2002). Palliative Care: the World Health Organization's global perspective. J Pain Symptom Manage.

[7] Organization WH (2007). Palliative Care.

[8] Quill TE, Abernethy AP (2013). Generalist plus specialist palliative care--creating a more sustainable model. N Engl J Med.

[9] Higginson IJ, Koffman J (2005). Public health and palliative care. Clin Geriatr Med.

[10] Bakitas M, Lyons KD, Hegel MT, Balan S, Brokaw FC, Seville J (2009). Effects of a palliative care intervention on clinical outcomes in patients with advanced cancer: the Project ENABLE II randomized controlled trial. JAMA.

[11] Casarett D, Pickard A, Bailey FA, Ritchie C, Furman C, Rosenfeld K (2008). Do palliative consultations improve patient outcomes?. J Am Geriatr Soc.

[12] Zimmermann C, Swami N, Krzyzanowska M, Hannon B, Leighl N, Oza A, et al. Early palliative care for patients with advanced cancer: a cluster-randomised controlled trial. Lancet. 2014 Feb 18. PubMed Epub 2014/02/25. Eng 10.1016/S0140-6736(13)62416-2 24559581

[13] Gade G, Venohr I, Conner D, McGrady K, Beane J, Richardson RH (2008). Impact of an inpatient palliative care team: a randomized control trial. J Palliat Med.

[14] Penrod JD, Deb P, Luhrs C, Dellenbaugh C, Zhu CW, Hochman T (2006). Cost and utilization outcomes of patients receiving hospital-based palliative care consultation. J Palliat Med.

[15] Temel JS, Greer JA, Muzikansky A, Gallagher ER, Admane S, Jackson VA (2010). Early palliative care for patients with metastatic non-small-cell lung cancer. N Engl J Med.

[16] Morrison RS, Penrod JD, Cassel JB, Caust-Ellenbogen M, Litke A, Spragens L (2008). Cost savings associated with US hospital palliative care consultation programs. Arch Intern Med.

[17] Ciemins EL, Blum L, Nunley M, Lasher A, Newman JM (2007). The economic and clinical impact of an inpatient palliative care consultation service: a multifaceted approach. J Palliat Med.

[18] May P, Normand C, Morrison RS. Economic Impact of Hospital Inpatient Palliative Care Consultation: Review of Current Evidence and Directions for Future Research. Journal of palliative medicine. 2014 Jul 1. PubMed Epub 2014/07/02. Eng. 10.1089/jpm.2013.0594 PMC4158953 24984168

[19] Field D, Addington-Hall J (1998). Extending specialist palliative care to all?. Soc Sci Med.

[20] Luddington L, Cox S, Higginson I, Livesley B (2001). The need for palliative care for patients with non-cancer diseases: a review of the evidence. Int J Palliat Nurs.

[21] Maciasz RM, Arnold RM, Chu E, Park SY, White DB, Vater LB (2013). Does it matter what you call it? A randomized trial of language used to describe palliative care services. Supportive care in cancer.

[22] Rainbird K, Perkins J, Sanson-Fisher R, Rolfe I, Anseline P (2009). The needs of patients with advanced, incurable cancer. Br J Cancer.

[23] Whitmer KM, Pruemer JM, Nahleh ZA, Jazieh AR (2006). Symptom management needs of oncology outpatients. J Palliat Med.

[24] Teunissen SC, Wesker W, Kruitwagen C, de Haes HC, Voest EE, de Graeff A (2007). Symptom prevalence in patients with incurable cancer: a systematic review. J Pain Symptom Manage.

[25] Beccaro M, Costantini M, Merlo DF (2007). Inequity in the provision of and access to palliative care for cancer patients. Results from the Italian survey of the dying of cancer (ISDOC). BMC Public Health.

[26] Nevadunsky NS, Gordon S, Spoozak L, Van Arsdale A, Hou Y, Klobocista M (2014). The role and timing of palliative medicine consultation for women with gynecologic malignancies: association with end of life interventions and direct hospital costs. Gynecol Oncol.

[27] Wentlandt K, Krzyzanowska MK, Swami N, Rodin GM, Le LW, Zimmermann C (2012). Referral practices of oncologists to specialized palliative care. J Clin Oncol.

[28] Lopez-Acevedo M, Lowery WJ, Lowery AW, Lee PS, Havrilesky LJ (2013). Palliative and hospice care in gynecologic cancer: a review. Gynecol Oncol.

[29] Ahmed N, Bestall JC, Ahmedzai SH, Payne SA, Clark D, Noble B (2004). Systematic review of the problems and issues of accessing specialist palliative care by patients, carers and health and social care professionals. Palliat Med.

[30] Kuin A, Courtens AM, Deliens L, Vernooij-Dassen MJ, van Zuylen L, van der Linden B (2004). Palliative care consultation in The Netherlands: a nationwide evaluation study. J Pain Symptom Manage.

[31] Teunissen SC, Verhagen EH, Brink M, van der Linden BA, Voest EE, de Graeff A (2007). Telephone consultation in palliative care for cancer patients: 5 years of experience in The Netherlands. Support Care Cancer.

[32] Grandjean I, Galesloot, C. Consultatie Palliatieve Zorg. Jaarverslag 2012. Integraal Kankercentrum Nederland (IKNL), 2013 September 2013. Report No.

[33] Galesloot CB-S, A.; Klinkenberg, M.; Van der Heide , A.; Baar, F.P.M. . Enquete stand van zaken palliatieve zorg in Nederlandse Ziekenhuizen. Verslag van een inventarisatie bij ziekenhuizen in Nederland door Stichting Leerhuizen Palliatieve Zorg, IKNL en het Erasmus MC. IKNL, Erasmus MC, Stichting Leerhuizen Palliatieve Zorg, 2013.

[34] Moss AH, Lunney JR, Culp S, Auber M, Kurian S, Rogers J (2010). Prognostic significance of the "surprise" question in cancer patients. J Palliat Med.

[35] Aaronson NK, Ahmedzai S, Bergman B, Bullinger M, Cull A, Duez NJ (1993). The European Organization for Research and Treatment of Cancer QLQ-C30: a quality-of-life instrument for use in international clinical trials in oncology. J Natl Cancer Inst.

[36] Pelayo-Alvarez M, Perez-Hoyos S, Agra-Varela Y (2013). Reliability and concurrent validity of the Palliative Outcome Scale, the Rotterdam Symptom Checklist, and the Brief Pain Inventory. J Palliat Med.

[37] Pickard AS, De Leon MC, Kohlmann T, Cella D, Rosenbloom S (2007). Psychometric comparison of the standard EQ-5D to a 5 level version in cancer patients. Med Care.

[38] Albers G, Echteld MA, de Vet HC, Onwuteaka-Philipsen BD, van der Linden MH, Deliens L (2010). Evaluation of quality-of-life measures for use in palliative care: a systematic review. Palliat Med.

[39] Balderas-Pena LM, Sat-Munoz D, Contreras-Hernandez I, Solano-Murillo P, Hernandez-Chavez GA, Mariscal-Ramirez I (2011). Evaluation of patient satisfaction with the quality of health care received within the EORTC IN-PATSAT32 trial by patients with breast and colorectal cancer, and non-Hodgkin lymphoma at different stages. Correlation with sociodemographic characteristics, comorbidities and other procedural variables at the Mexican Institute of Social Security. Value Health.

[40] Bredart A, Bottomley A, Blazeby JM, Conroy T, Coens C, D'Haese S (2005). An international prospective study of the EORTC cancer in-patient satisfaction with care measure (EORTC IN-PATSAT32). Euro J Cancer (Oxford, England : 1990).

[41] Rosenbaum PR (1984). Reducing bias in observational studies. J Am Stat Assoc.

[42] Griswold ME, Localio AR, Mulrow C (2010). Propensity score adjustment with multilevel data: setting your sites on decreasing selection bias. Ann Intern Med.

[43] Austin PC, Mamdani MMA (2006). comparison of propensity score methods: a case-study estimating the effectiveness of post-AMI statin use. Stat Med.

[44] Gold MR eae. Cost-Effectiveness in Health and Medicine. New York: Oxford University Press; 1996.

[45] Hakkaart-van Roijen L ea. Handleiding voor kostenonderzoek, methoden en standaard kostprijzen voor economische evaluaties in de gezondheidszorg. College voor zorgverzekeringen., Geactualiseerde versie, 2010.

[46] Williams CJ, Shuster JL, Clay OJ, Burgio KL (2006). Interest in research participation among hospice patients, caregivers, and ambulatory senior citizens: practical barriers or ethical constraints?. J Palliat Med.

[47] Vandenbroucke JP (2004). When are observational studies as credible as randomised trials?. Lancet.

[48] LeBlanc TW, Wheeler JL, Abernethy AP (2010). Research in end-of-life settings: an ethical inquiry. J Pain Palliat Care Pharmacother.

[49] Prioritising palliative care. Lancet. 2014 May 17;383(9930):1694. PubMed Epub 2014/05/20. eng. 10.1016/S0140-6736(14)60814-X 24835602

